# Microbial indicators of environmental perturbations in coral reef ecosystems

**DOI:** 10.1186/s40168-019-0705-7

**Published:** 2019-06-21

**Authors:** Bettina Glasl, David G. Bourne, Pedro R. Frade, Torsten Thomas, Britta Schaffelke, Nicole S. Webster

**Affiliations:** 1 Australian Institute of Marine Science, Townsville, QLD Australia; 2 College of Science and Engineering, James Cook University, Townsville, QLD Australia; 3 AIMS@JCU, Townsville, QLD Australia; 4 Centre of Marine Science, University of Algarve, Faro, Portugal; 5 Centre for Marine Bio-Innovation & School of Biological, Earth and Environmental Sciences, University of New South Wales, Sydney, Australia; 6 Australian Centre for Ecogenomics, University of Queensland, Brisbane, QLD Australia

**Keywords:** Microbial monitoring, Coral reef, Machine learning, Microbial indicators, Coral reef microbiomes, Microbial baselines

## Abstract

**Background:**

Coral reefs are facing unprecedented pressure on local and global scales. Sensitive and rapid markers for ecosystem stress are urgently needed to underpin effective management and restoration strategies. Although the fundamental contribution of microbes to the stability and functioning of coral reefs is widely recognised, it remains unclear how different reef microbiomes respond to environmental perturbations and whether microbiomes are sensitive enough to predict environmental anomalies that can lead to ecosystem stress. However, the lack of coral reef microbial baselines hinders our ability to study the link between shifts in microbiomes and ecosystem stress. In this study, we established a comprehensive microbial reference database for selected Great Barrier Reef sites to assess the diagnostic value of multiple free-living and host-associated reef microbiomes to infer the environmental state of coral reef ecosystems.

**Results:**

A comprehensive microbial reference database, originating from multiple coral reef microbiomes (i.e. seawater, sediment, corals, sponges and macroalgae), was generated by 16S rRNA gene sequencing for 381 samples collected over the course of 16 months. By coupling this database to environmental parameters, we showed that the seawater microbiome has the greatest diagnostic value to infer shifts in the surrounding reef environment. In fact, 56% of the observed compositional variation in the microbiome was explained by environmental parameters, and temporal successions in the seawater microbiome were characterised by uniform community assembly patterns. Host-associated microbiomes, in contrast, were five-times less responsive to the environment and their community assembly patterns were generally less uniform. By applying a suite of indicator value and machine learning approaches, we further showed that seawater microbial community data provide an accurate prediction of temperature and eutrophication state (i.e. chlorophyll concentration and turbidity).

**Conclusion:**

Our results reveal that free-living microbial communities have a high potential to infer environmental parameters due to their environmental sensitivity and predictability. This highlights the diagnostic value of microorganisms and illustrates how long-term coral reef monitoring initiatives could be enhanced by incorporating assessments of microbial communities in seawater. We therefore recommend timely integration of microbial sampling into current coral reef monitoring initiatives.

**Electronic supplementary material:**

The online version of this article (10.1186/s40168-019-0705-7) contains supplementary material, which is available to authorized users.

## Background

Coral reef ecosystems are rapidly degrading due to local and global pressures [1]. Overfishing, pollution, declining water quality, disease and outbreaks of coral predating crown-of-thorns starfish are responsible for localised reef degradation [2] while climate change is impacting reefs on a global scale, including remote reefs with little local anthropogenic pressure [3]. For example, elevated sea surface temperatures caused back-to-back coral mass bleaching events in 2016 and 2017, resulting in a significant loss of shallow-water corals on the Great Barrier Reef (GBR) [4]. Climate conditions predicted for the end of the century will result in even more frequent and severe coral mass bleaching events with dire projections for the future of coral reefs [5, 6]. This global coral reef crisis is driving the development of new management, reef restoration and bioengineering tools to counteract reef loss and ensure the persistence of coral reefs [7, 8]. Early prediction of ecosystem stress is critical for an effective implementation of local management and restoration strategies on threatened reef sites.

Microorganisms have considerable potential as a monitoring tool for coral reef ecosystem health [9–11]. Microorganisms are fundamental drivers of biogeochemical cycling on coral reefs [12–14]; they form intimate associations with the coral reef benthos [15–17], and they contribute significantly to host health and ecosystem homeostasis [18–20]. The constant amendment of microbial communities to exploit available resources [21] can trigger differential abundances of specific microorganisms; hence, shifts in community composition can provide an early indication of environmental change [22]. For example, compositional and functional shifts of coral-associated microbial communities have been described along gradients of anthropogenic impact [23–25] and with changes in water quality [26]. However, despite having many of the useful characteristics required of environmental indicators [9, 27], the diagnostic potential of microorganisms for coral reef monitoring is largely conceptual, with only a few studies elaborating on their potential value. For example, the ‘microbialisation score’ measures human impacts on coral reefs based on the ratio of microbial and fish metabolic rates [28]. The main limitations to further develop and apply microbial-based monitoring approaches are the lack of temporal and spatial baselines for coral reef microbiomes [9, 29].

Coral reefs comprise a complex network of free-living and host-associated microbial communities with strong benthic-pelagic exchange [13, 30]. Therefore, holistic assessments that combine different reef hosts and habitats are required to better understand microbial dynamics and sensitivities to environmental perturbations. The diagnostic value of microbial-based monitoring is likely to vary between distinct habitats of a coral reef ecosystem. For example, microbial communities occurring in seawater may be directly affected by the quality of the ambient reef water or climate conditions; however, the high heterogeneity of seawater due to local hot-spots of available resources [31, 32] may diminish the specificity of these communities. In contrast, microbial communities that dwell in corals live in tight association with the most important frame-builders of reefs [29] and hence may provide crucial information not only on the environmental conditions but also on the effect of the environment on the coral host itself. Sponges, a highly abundant and diverse component of coral reefs [33], are renowned for their enormous filtration capacity [34] and form diverse and intimate associations with microbial communities [35]. Hence, sponge microbiomes may provide suitable indicators to monitor water quality. Host-associated biofilms, such as those inhabiting the mucus layer of corals and the surface of macroalgae, provide another potential niche habitat informative for microbial indicators of environmental state. Coral mucus, for example, has been described as a suitable habitat to screen for enterobacteria from sewage contamination due to its ability to trap bacteria [36].

Given the complexity of microbial life on coral reefs, we sought to identify the most suitable reef microbiomes for a microbial indicator program to pinpoint environmental state. To do this, we quantified the (1) habitat-specificity, (2) determinacy of microbial community successions and (3) sensitivity towards environmental parameters of multiple free-living and host-associated microbiomes. Subsequently, we tested the microbiome’s ability to infer environmental state using indicator value [37] and machine learning approaches [38].

## Results

**Fig. 1:**
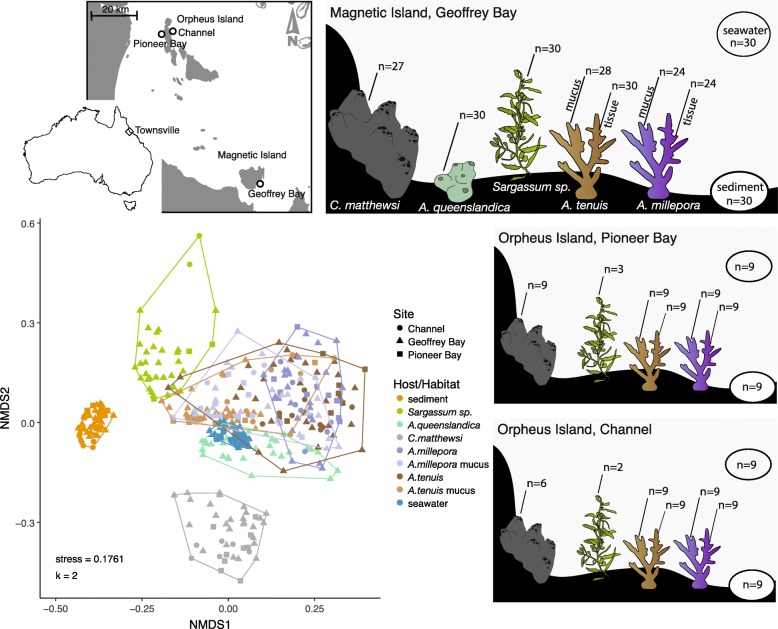
Habitat-specificity of coral reef microbiomes. Seawater, sediment, coral (*Acropora tenuis* and *Acropora millepora*), sponge (*Amphimedon queenslandica* and *Coscinoderma matthewsi*) and macroalgae (*Sargassum* sp.) samples were collected for 16S rRNA gene sequencing at fringing reefs surrounding Magnetic Island (Geoffrey Bay) and Orpheus Island (Pioneer Bay and Channel; Queensland, Australia). Non-metric multidimensional scaling (NMDS) based on Bray-Curtis dissimilarities revealed high habitat-specificity of coral reef microbiomes

Samples were collected during a 16-month period (February 2016–May 2017), at monthly (Magnetic Island—Geoffrey Bay) and periodic (Orpheus Island—Pioneer Bay and Channel) intervals (Additional file 1: Table S1). The bacterial 16S rRNA genes of 381 samples including seawater, sediment, sponge tissue (*Coscinoderma matthewsi* and *Amphimedon queenslandica*), coral tissue and mucus (*Acropora tenuis* and *Acropora millepora*) and macroalgal surfaces (*Sargassum* sp.) were sequenced (Fig. 1). In total 231,316 zero-radius operational taxonomic units (zOTUs) were identified based on 100% sequence similarity [39].

### Coral reef microbiomes are habitat-specific

Habitat-specificity of coral reef microbes was assessed by comparing the similarities of microbial communities associated with seawater (*n* = 48), sediment (*n* = 48), *A*. *queenslandica* (*n* = 30), *C*. *matthewsi* (*n* = 42), *A*. *tenuis* (tissue *n* = 48, mucus *n* = 46*)*, *A*. *millepora* (tissue *n* = 42, mucus *n* = 42) and *Sargassum* sp. (*n* = 35). Non-metric multidimensional scaling based on Bray-Curtis dissimilarities revealed a clear separation of the microbial communities from different reef habitats (Fig. 1), and habitat-specificity was further confirmed with permutational multivariate analysis of variance (PERMANOVA, *p* = 9.999 × 10^−5^, Additional file 1: Tables S2-S3). Furthermore, alpha diversities (ANOVA, *F*_(8/372)_ = 142, *p* = 2 × 10^−16^) and zOTU richness (ANOVA, *F*_(8/372)_ = 369, *p* = 2 × 10^−16^) varied significantly between reef habitats (Additional file 1: Figure S1 and Tables S4-S6). Sediment harboured by far the most diverse (Shannon Index 7.4 ± 0.2 SD) bacterial community, although microbial diversity was also high in coral surface mucus (Shannon Index 5.1 ± 0.9 SD), macroalgal biofilms (Shannon Index 4.5 ± 1.4 SD), seawater (Shannon Index 4.4 ± 0.2 SD) and in the tissue of the sponge *C*. *matthewsi* (Shannon Index 4.4 ± 0.3 SD). Microbial diversity was lowest in coral tissue (Shannon Index 3.3 ± 0.8 SD) and in the sponge *A*. *queenslandica* (Shannon Index 2.7 ± 0.8 SD). These results suggest overall high habitat-specificity of free-living and host-associated microbial communities within coral reef ecosystems.

### Uniform vs variable community assembly patterns

**Fig. 2:**
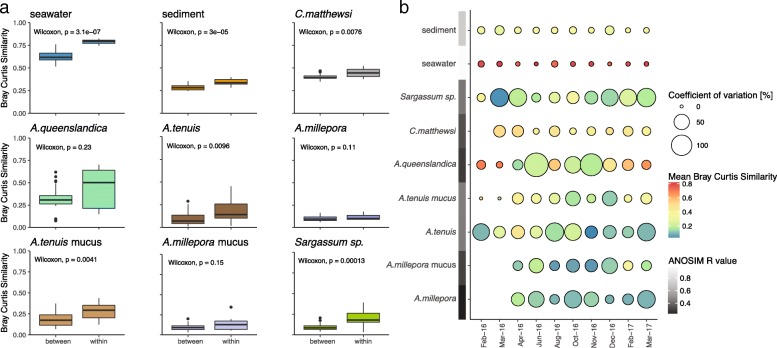
Compositional similarity of coral reef microbiomes over time. **a** Variations in the compositional similarity between and within sampling time points of various coral reef microbiomes collected at Geoffrey Bay (Magnetic Island). A higher similarity within time point replicates than between time point replicates suggests a uniform response of the microbial community to temporal variations. Similarities were calculated with Bray-Curtis Similarity Index (0 = no similarity, 1 = high similarity) and significances tested with Wilcoxon rank-sum test. **b** The within sampling time point similarities of replicates (*n* = 3) is indicated in colour and the dispersion (coefficient of variation—ratio of the standard deviation to the mean expressed as percentage) is displayed as size. Analysis of similarity (ANOSIM) was further used as a proxy for the within and between time point variation. *R* values of 1 indicate high similarity within sampling time points and high variability between sampling time points, whereas 0 indicates equal similarity within and between sampling time points

The uniformity versus variability of microbial community assembly patterns was explored through comparison of compositional similarity (Bray-Curtis Similarity Index, 0 = dissimilar, 1 = identical) in samples collected monthly at Geoffrey Bay (Magnetic Island). The microbial communities of seawater (*n* = 30, Wilcoxon Rank-Sum test *p* = 3.1 × 10^−7^) and sediment (*n* = 30; Wilcoxon Rank-Sum test *p* = 3 × 10^−5^) had significantly higher similarities ‘within’ than ‘between’ sampling events (Fig. 2a). This uniform response of the free-living microbial communities suggests that deterministic rather than stochastic processes drive their community assembly. For host-associated microbiomes, the overall response pattern varied between species. Microbial communities associated with the sponge *C*. *matthewsi* (*n* = 27; Wilcoxon Rank-Sum test, *p* = 0.0076), the coral *A*. *tenuis* (mucus *n* = 28, tissue *n* = 30; Wilcoxon Rank-Sum test, *p* = 0.0041 and *p* = 0.0096, respectively) and the macroalga *Sargassum* sp. (*n* = 30; Wilcoxon Rank-Sum test, *p* = 0.00013) followed the same trend as the free-living communities, with significantly higher similarities ‘within’ than ‘between’ sampling events (Fig. 2a). In contrast, the microbiome of the sponge *A*. *queenslandica* (*n* = 30; Wilcoxon Rank-Sum test, *p* = 0.23) and the coral *A*. *millepora* (mucus *n* = 24, tissue *n* = 24; Wilcoxon Rank-Sum test, *p* = 0.15 and *p* = 0.11 respectively) showed no significant difference in similarities ‘within’ and ‘between’ time points (Fig. 2a). Analysis of the compositional similarity of sample replicates within each sampling time point indicated that the seawater microbial communities not only exhibit an overall higher similarity ‘within’ replicates, but the high compositional similarity is conserved across all sampling events (Fig. 2b). In contrast, host-associated microbial communities showed a generally lower compositional similarity and higher variation between sample replicates within each sampling time point (Fig. 2b).

Trends in the temporal community assembly pattern of free-living, host tissue- and biofilm-associated microbial communities were analysed using analysis of similarity (ANOSIM) as a proxy to describe similarity patterns (*R* = 0 indicates equal similarity ‘within’ and ‘between’ time point replicates and *R* = 1 indicates higher ‘within’ than ‘between’ sampling time point similarities; Fig. 2b and Additional file 1: Figure S2). Overall, free-living microbiomes had *R* values closer to 1 (seawater *R* = 0.9919 and sediment *R* = 0.7322), whereas host-associated microbiomes had *R* values closer to 0 (*A*. *queenslandica R* = 0.2927, *C*. *matthewsi R* = 0.3449, *A*. *tenuis* tissue *R* = 0.4547, *A*. *millepora* tissue *R* = 0.2151, *A*. *tenuis* mucus *R* = 0.4613, *A*. *millepora* mucus *R* = 0.3090 and *Sargassum* sp. biofilm *R* = 0.4440; Fig. 2b and Additional file 1: Figure S2). These results suggest that free-living microbiomes (seawater and sediment) exhibit a uniform compositional succession, whereas host-associated microbiomes (coral, sponge and macroalgae) are more stochastic in their temporal community succession. The uniform temporal response of free-living microbiomes suggests a high diagnostic value of these microbial communities; hence seawater and sediment microbiomes should provide an accurate prediction of environmental variables.

Microbiomes in seawater (*n* = 48) and sediment (*n* = 48) were further tested for their compositional similarity between all three sampling sites (Geoffrey Bay, Pioneer Bay and Channel). The microbial community composition of sediment samples varied significantly between sampling sites (ANOSIM R = 0.9430, *p* = 0.001, Additional file 1: Figure S3a). The seawater microbiome, in contrast, showed high temporal variability (ANOSIM *R* = 0.9934, *p* = 0.001) and low spatial variability (ANOSIM *R* = 0.2343, *p* = 0.002; Additional file 1: Figure S3b). The high spatial variability of sediment microbiomes indicates that habitat characteristics rather than environmental fluctuations are the main drivers structuring community composition.

### Environmental sensitivity

**Fig. 3:**
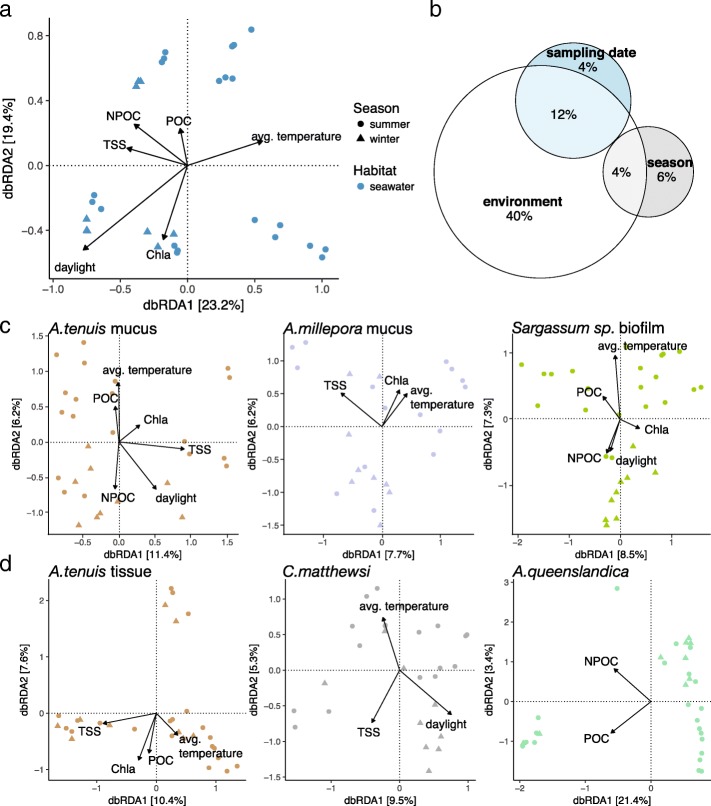
Coral reef microbiome sensitivity to environmental parameters. Bray-Curtis distance-based RDA (dbRDA) was used to evaluate the effect of environmental fluctuations on the microbial community composition of various coral reef habitats/hosts. The total variance (in percent) explained by each axis is indicated in parentheses. **a** Environmental factors (average temperature, daylight, TSS, NPOC, Chl *a* and POC) significantly explained the observed compositional variation in the seawater-associated microbial community (permutational ANOVA for dbRDA). **b** Variation partitioning shows that environmental parameters (average temperature, daylight, TSS, NPOC, Chl *a* and POC) rather than season and/or sampling date explain observed community composition structures in the seawater microbiome. **c** Coral mucus and algae biofilm as well as **d** coral and sponge tissue microbial communities were significantly influenced by environmental factors; however, environmental parameters only explain on average 11% of the observed community variation (Additional file 1: Table S7)

Environmental sensitivity of the different microbiomes was assessed by comparing how much of the compositional variation was explained by sea surface temperature, light and water quality parameters (Additional file 1: Figures S4 and S5). The compositional variability of the seawater microbiome (*n* = 30) was significantly explained by sampling date, season (summer *versus* winter) and water quality parameters, such as average seawater temperature, average hours of daylight, total suspended solids (TSS), particulate organic carbon (POC), chlorophyll *a* (Chl *a*) and non-purgeable organic carbon (NPOC) concentration (permutational ANOVA for Bray Curtis distance-based redundancy analysis (dbRDA); Fig. 3a and Additional file 1: Table S7a-b). In total, these environmental parameters explained 56% of the observed compositional variation in seawater (variation partitioning analysis, Fig. 3b, Additional file 1: Table S7). Season (summer versus winter) and sampling date solely explained 6% and 4%, respectively (variation partitioning analysis, Fig. 3b). In comparison, sampling site significantly explained 24% of the variation in sediment microbial communities (*n* = 48), which overlapped by 12% with the variation explained by sediment characteristics, such as particle size and total organic carbon (TOC) content (permutational ANOVA for dbRDA and variation partitioning analysis; Additional file 1: Tables S7b and S8). Water quality parameters and sea surface temperature explained only 3% of the observed variability in the sediment microbiome (variation partitioning analysis).

Host-associated microbiomes varied substantially in their response to environmental parameters (permutational ANOVA for dbRDA and variation partitioning analysis, Fig. 3b, c, Additional file 1: Tables S7c-i and S8). On average, 11% of the observed community variations in host-associated microbiomes were explained by the environment (variation partitioning analysis), which is five times less than what we found for the seawater-associated microbial community (Additional file 1: Table S8). This suggests that compositional variations of the seawater microbiome are more likely to reflect environmental changes. Host-associated microbiomes are comparatively stable to changes in environmental factors.

### Predictability of environmental metadata

**Fig. 4:**
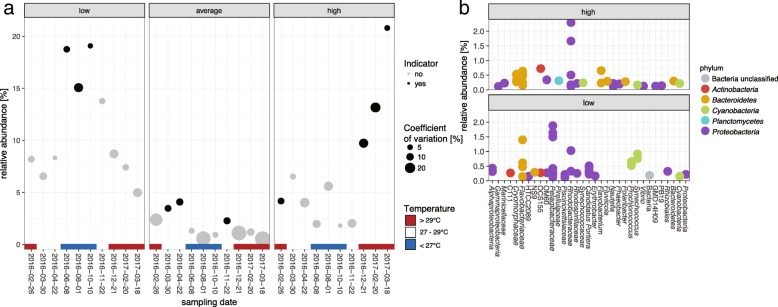
Microbial indicator taxa for seawater temperature fluctuations. Seawater temperatures were z-score standardised and, based on the variation around their mean, classified into low (< − 0.5), average (− 0.5–0.5) and high (> 0.5) temperature groups. Indicator zOTUs were identified with the indicator value analysis (IndVal). **a** The average relative abundance of the sum of low, average and high temperature indicators is represented for each sampling time point. Significant indicator zOTUs assemblages (*p* < 0.01) for the respective temperature group are indicated in black and size represents the coefficient of variation. Colour gradient further represents the seawater temperature at the given sampling timepoints. **b** Relative abundances and taxonomic affiliation of zOTUs identified to be significant (*p* < 0.01) indicators for high and low seawater temperatures. Each dot represents a unique zOTU

Due to the seawater microbiomes uniform temporal pattern and high sensitivity to changing environmental parameters, the ability to infer environmental state based on microbial community data was tested using an indicator value analysis [37] and a random forest machine learning approach. In total, 110 zOTUs were identified as significant indicators for temperature (indicator value *p* < 0.01). Microbial zOTU assemblages that were indicative of high, low and average seawater temperatures (classification based on their variation around observed annual averages) were present throughout the sampling period. However, higher relative abundances and lower variation (as calculated by coefficient of variation) were evident at certain time points (Fig. 4a). Furthermore, we were able to identify microbial indicator taxa for high and low Chl *a*, TSS and POC levels (Additional file 1: Figure S6). Indicators for low and high seawater temperatures were identified in the bacterial phyla *Proteobacteria*, *Bacteroidetes*, *Cyanobacteria*, *Actinobacteria* and *Planctomycetes* (Fig. 4b). High temperatures were indicated by an increase of zOTUs belonging to the bacterial family *Rhodobacteraceae* and the presence of *Cryomorphaceae*, *Synechococcaeae*, *Vibrio* and *Flavobacterium* (Fig. 4b). In contrast, the occurrence of zOTUS belonging to the family *Pelagibacteriaceae* and the genus *Prochlorococcus* were indicative for low seawater temperatures. The phyla *Proteobacteria*, *Bacteroidetes* and *Cyanobacteria* had the greatest number of indicator zOTUs for temperature and other water quality parameters (Additional file 1: Figure S6). *Flavobacteriaceae*-affiliated zOTUs were significant indicators for temperature, Chl *a*, TSS and POC. *Halomonadaceae* significantly associated with high Chl *a* and TSS and zOTUs belonging to the phylum *Verrucomicrobia* were significant indicators for high TSS levels.

**Fig. 5:**
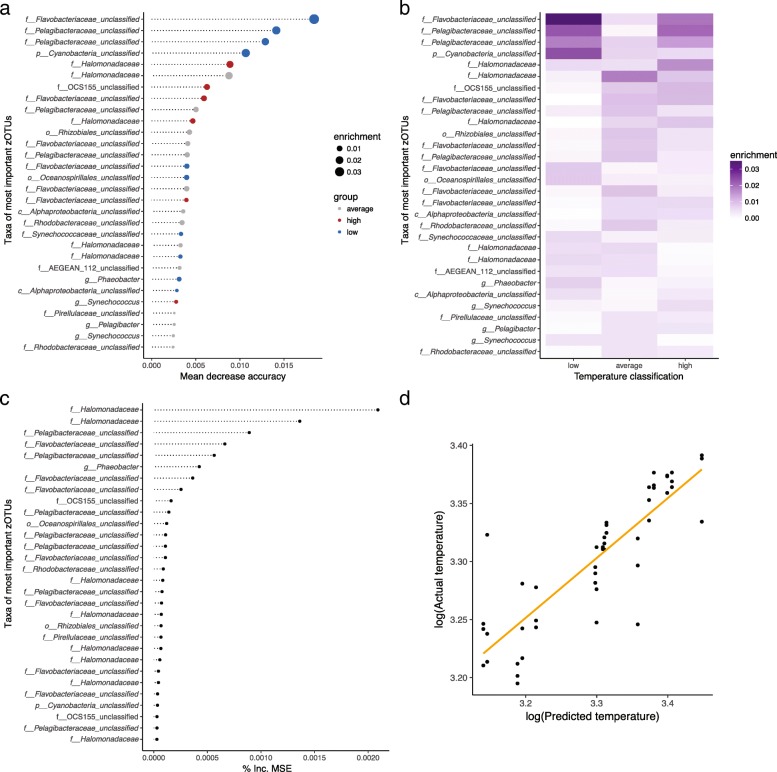
Random forest machine learning. **a** The 30 most important zOTUs reducing the uncertainty in the prediction of seawater temperature classes (low, average, high) based on their mean decrease in accuracy and **b** their enrichment in the temperature classes. **c** The 30 most important zOTUs reducing the variance (mean squared error (% Inc. MSE)) in regression-based prediction of seawater temperatures. **d** Predicted seawater temperature values versus actual seawater temperature values based on random forest regression

The diagnostic value of the seawater microbiome (*n* = 48) was further evaluated by applying a random forest machine learning classification and regression analysis with 1213 zOTUs preselected based on a non-zero abundance threshold in at least 10% of the samples (*n* = 48). The seawater microbiome enabled the prediction of seawater temperature classes (low, average, high) with 92% accuracy (Kappa = 88%, Fig. 5a, b and Additional file 1: Figure S7). Highest accuracy (lowest out of bag (OOB) estimated error rate) was achieved with *m*_try_ = 100 zOTUS. Random forest regression of the seawater microbiome predicted temperature values (*R*^2^ = 0.67, RMSE = 0.5) (Fig. 5c, d and Additional file 1: Figure S8) with the highest accuracy (lowest OOB estimated error rate) when *m*_try_ = 400 zOTUs. The effectiveness of zOTUs in reducing uncertainty and variance (also referred to as ‘feature importance’) within the machine learning algorithm was measured by the decrease in mean accuracy for classification and mean-squared error (% Inc. MSE) for regression. The most important zOTUs belong to the bacterial taxa *Flavobacteriaceae*, *Pelagibacteraceae*, *Cyanobacteria*, *Rhodobacteraceae*, *Synechococcaceae* and *Pirrelulaceae*. These results demonstrate that the microbial community associated with coral reef seawater allows for the accurate prediction of fluctuations in sea surface temperature and water quality parameters.

## Discussion

Sensitive and rapidly responding markers of coral ecosystem stress are needed to underpin effective management and restoration strategies. In this study, we used a range of statistical tests and machine learning approaches across multiple free-living and host-associated reef microbiomes to assess their diagnostic value as sensitive indicators of environmental state. Our results show that the microbial community in reef seawater has the highest diagnostic value when compared to other free-living (e.g. sediment) and host-associated microbiomes (e.g. coral, sponge and macroalgae). Our conclusion is based on the microbiome’s (1) habitat-specificity, (2) uniformity of its community assembly, (3) sensitivity towards environmental fluctuations and (4) accuracy to predict environmental parameters. This assessment of the diagnostic capacity of various free-living and host-associated coral reef microbiomes to extrapolate environmental variations provides crucial information for ecosystem management initiatives aimed at incorporating microbial monitoring.

In general, high habitat-specificity was observed across free-living and host-associated microbiomes, confirming previous reports on the compositional variability of microbial communities between coral reef habitats [40], host species [15, 41–43] and even between host compartments [44]. High compositional divergence of microbial communities across different reef habitats can be due to the variation of available resources and/or biotic interactions [21]. High habitat-specificity contributes to the overall high diversity and complexity across different microbial communities on coral reefs, highlighting the importance of holistic studies that focus on microbial interactions across the benthic-pelagic realm.

Bacterial community structure associated with water and sediment is thought to be primarily governed by deterministic processes [45]. Our results are consistent with this, showing uniform community assembly patterns within time point replicates. In contrast, host-associated microbiomes displayed little compositional similarity within a sampling time point, suggesting a non-uniform temporal response. Host-associated microbiomes were also only marginally affected by environmental parameters, indicating that their community assembly pattern is variable between conspecific individuals [45]. A higher variability in community assembly can lead to increased community heterogeneity, also referred to as dispersion, which has been described as a common characteristic of host-associated microbiomes [18, 46–48]. Furthermore, lower microbial compositional similarities among replicates may be driven by increased niche space (e.g. host compartments) [44] and host genotype effects (e.g. host genetics) [42]. Collectively, our results show that free-living microbial communities have a higher potential to infer environmental parameters (such as standard measures in environmental monitoring programs) than host-associated microbial communities due to their higher uniformity and environmental sensitivity. Importantly however, previous metaproteomic research on reef sponges has shown that while microbial community composition can appear stable when seawater temperatures increase, disruption to nutritional interdependence and molecular interactions (such as reduced expression of transporters involved in the uptake of sugars, peptides and other substrates) actually occurs prior to detectable changes in community structure [49]. Hence, considering the importance of microbes to reef invertebrate health, more sensitive transcriptomic/proteomic approaches may still be warranted for sensitive detection of microbial responses to environmental perturbations.

The diagnostic potential of microbial communities, especially in combination with machine learning approaches, has gained momentum across multiple research fields, including disease identification by characterisation of the human gut-microbiome [50], evaluation of the environment and host genetics on the human microbiome [51], prediction of hydrological functions in riverine ecosystems [52] and assessment of macroecological patterns in soil samples [53]. This development of microbial-based diagnostics is largely due to availability of high-throughput sequencing of the 16S rRNA gene and streamlined analytical pipelines that facilitate rapid assessment of microbial community composition [54, 55]. In addition to its utility for inferring environmental fluctuations, the seawater microbiome possesses numerous characteristics desirable for environmental monitoring programs: (i) non-destructive collection and simple processing methods facilitate large-scale collections alongside existing programs that sample water quality measurements, (ii) high fractional contribution of abundant microbes minimises the impacts of sequencing biases (Additional file 1: Figure S9) and (iii) sampling is conducive to future automated, high throughput analyses such as in-line flow cytometry on vessels and real-time DNA/RNA sequencing for community characterisation.

Incorporation of seawater microbial community data into coral reef monitoring approaches should enhance our ability to describe environmental conditions and changes more holistically. For example, temperature fluctuations drive structural variations in seawater microbial communities [56, 57], and elevated seawater temperatures on coral reefs are highly correlated with coral bleaching [1, 58]. The inclusion of microbial community data alongside water quality parameters could therefore improve our ability to predict the likelihood of ecosystem stress. For instance, our sample sites, located in the central sector of the GBR, were not affected by the 2016 bleaching that primarily affected the northern sector [59]; however, they were impacted by the 2017 bleaching event [60]. In the months prior to bleaching (late December 2016 till March 2017), we observed two to four times higher relative abundances of high temperature indicator assemblages than when compared to the equivalent period at the beginning of 2016 (Fig. 4a), where no bleaching was observed. Interestingly, high temperature indicator assemblages included putative coral pathogens (e.g. *Vibrio*) and opportunistic bacteria (e.g. *Rhodobacteraceae*, *Verrucomicrobia* and *Flavobacterium*). Coral pathogens, such as *Vibrio corallilyticus*, increase their efficiency and motility behaviours with rising seawater temperatures [61–63], and the higher abundance of these microbes may explain the increased prevalence of coral disease post bleaching [64]. Hence, microbial monitoring could help inform managers about impending disease outbreaks.

## Conclusion

Our study provides the first holistic microbial baseline spanning multiple free-living and host-associated microbiomes for selected GBR sites. Results suggest that there is realistic scope to enhance long-term reef monitoring initiatives by incorporating seawater microbiome observations for assessments of environmental change over space and time, especially for rapid and sensitive identification of early signs of declining ecosystem health. The establishment of microbial observatories [65] and DNA biobanks for long-term biomonitoring [66] will be paramount to successfully inferring ecosystem state and/or perturbations from microbial communities. We therefore recommend timely integration of microbial sampling into current coral reef monitoring initiatives. Further refinement of the sampling and data analysis techniques should focus on selection and validation of additional indicator taxa as well as assessment of ecologically important microbial functions. A further consideration is to explore which monitoring objectives would benefit most from assessments of microbial communities. For example, it is likely that the rapid response time of microbial indicators makes them better suited to early-warning, impact or compliance monitoring programs than to monitoring of slower, long-term changes.

## Methods

### Sample collection

Samples for microbial community characterisation were collected monthly (Magnetic Island) and periodically (Orpheus Island) from seawater, sediment and multiple host organisms (i.e. corals, sponges and macroalgae), along with environmental metadata, between February 2016 and May 2017 at three Great Barrier Reef sites (Fig. 1). Samples were collected under the permit G16/38348.1 issued by the Great Barrier Reef Marine Park Authority.

Samples (*n* = 3/sample type/sampling event) for molecular analysis and additional environmental metadata were collected following the standard operational procedures of the Australian Marine Microbial Biodiversity Initiative (AMMBI; https://data.bioplatforms.com/organization/pages/australian-microbiome/methods). In brief, seawater for molecular analysis was collected with collapsible sterile bags close to the reef substrate at 2 m depth and pre-filtered (50 μm) to remove large particles and subsequently filtered (2 L) onto 0.2 μm Sterivex-filters (Millepore). The sediment surface layer was sampled with sterile 50 mL tubes at 2 m depth and subsampled immediately into 2 mL cryogenic vials. The sponges *Coscinoderma matthewsi* and *Amphimedon queenslandica* were removed from the substrate (at 7 m and 3 m respectively) with sterile scalpel blades, rinsed with 0.2 μm filter-sterilised seawater and subsampled into 2 mL cryogenic vials. The surface mucus layer of the two acroporid coral species, *Acropora tenuis* and *Acropora millepora*, was sampled with sterile cotton swabs [18]. Additionally, coral fragments of each sampled coral were collected at 3 m depth. Coral fragments were rinsed with 0.2 μm filtered-sterilised seawater and placed into 5 mL cryogenic vials. The thallus (including stem, floats and blades) of the macroalgae *Sargassum* sp. was sampled with sterile scalpels at 3 m depth, rinsed with 0.2 μm filtered-sterilised seawater and placed into 2 mL cryogenic vials. All samples were immediately flash frozen in liquid nitrogen after processing and stored at − 80 °C until DNA extraction.

Additional seawater samples were collected with a diver-operated Niskin bottle close to the reef substrate at 2 m depth at each sampling occasion. Water was subsampled in duplicate for analyses of salinity and concentrations of dissolved organic carbon (DOC), dissolved inorganic carbon (DIC), particulate organic carbon (POC), dissolved inorganic nutrients (DIN), total suspended solids (TSS) and chlorophyll a (Chl a) concentration. Samples were further analysed according to the standard procedures of the Australian Institute of Marine Science (AIMS, Townsville, Australia) [67]. Sediment samples were collected with 100 mL glass jars at 2 m depth and characteristics, such as grain size distribution and total organic carbon (TOC) and nitrogen (TON) content, were assessed for each sampling event. Seawater temperatures were obtained from AIMS long-term monitoring temperature records (http://eatlas.org.au/).

### DNA extraction

Prior to extraction, the macroalgal biofilm was separated from the algal tissue by overnight incubation at 200 rpm in 10 mL 1x PBS at 37 °C. Coral fragments were defrosted on ice and the tissue was stripped from the skeleton with an airgun into 1x PBS solution, homogenised for 1 min at 12.5 rpm with a tissue homogeniser, pelleted (10 min at 16,000 rcf) and snap frozen in liquid nitrogen prior to DNA extraction. DNA from seawater, sediment, sponge and macroalgal biofilms was extracted with the DNeasy PowerSoil kit (Qiagen) and DNA of coral tissue and mucus samples was extracted using the DNeasy PowerBiofilm kit (Qiagen) following the Manufacturer’s instructions. DNA extracts were stored at − 80 °C until being sent for sequencing.

### 16S rRNA gene sequencing

DNA extracts were sent on dry ice to the Ramaciotti Centre for Genomics (Sydney, Australia) for sequencing. The bacterial 16S rRNA genes were sequenced using the 27F [68] and 519R [69] primer pairs on the Illumina MiSeq platform utilising a duel indexed 2 × 300 bp paired end approach. Further documentation outlining the standard operating procedures for generating and sequencing amplicons is available at https://data.bioplatforms.com/dataset/marine-microbes-methods.

### Sequence analysis

Sequencing data were analysed as single nucleotide variants in a standardised platform alongside other Australian microbial biodiversity initiative samples [39, 70]. In brief, forward and reverse reads were merged using FLASH [71]. FASTA formatted sequences were extracted from FASTQ files and those < 400 bp in length or containing N’s or homopolymer runs of > 8 bp were removed using MOTHUR (v1.34.1) [72]. USEARCH (64 bit v10.0.240) [73] package was used to de-replicate sequences and to order them by abundance. Sequences with < 4 representatives and Chimeras were removed. Quality-filtered sequences were mapped to chimera-free zero-radius operational taxonomic units (zOTUs) and a sample by read abundance table created. zOTUs were taxonomically classified with SILVA v132 [74] database using MOTHUR’s implementation of the Wang classifier [75] and a 60% Bayesian probability cut-off.

Chloroplast and mitochondria-derived reads as well as singletons were removed from the dataset. Remaining data were rarefied to 3600 reads per sample and transformed to relative abundances using the phyloseq package [76] in R [77].

### Habitat and host-specificity

Habitat and host-specificity of a microbiome was assessed by calculating the compositional similarities of all 381 samples with the Bray-Curtis Similarity Index and illustrating them in a non-metric multidimensional scaling (NMDS) plot using the phyloseq package [76]. To confirm habitat and host-specificity, permutational multivariate analysis of variance (PERMANOVA) was applied using the adonis() function of the vegan package [78] with 10,000 permutations.

### Uniform response pattern

The microbiome similarity of replicates for sampling time points versus the microbiome similarity between sampling time points was compared by obtaining the Bray-Curtis Similarity for each habitat individually. The variation between the overall within and between time point replicates was tested with a Wilcoxon Rank-sum test in R [77]. The dispersion of the Bray-Curtis Similarities within a sampling time point was calculated as the coefficient of variation (ratio of the standard deviation to the mean expressed as a percentage). The higher the coefficient of variation, the higher the variability in the microbiome composition among replicates of a time point. Analysis of similarity (ANOSIM; anosim() function of the vegan package [78]) based on Bray-Curtis Similarities was used to further evaluate within and between time point similarities in the microbial communities.

### Environmental sensitivities

Environmental metadata were z-score standardised [79] and checked for collinearity using the Pearson correlation coefficient. Collinearity was assumed if correlation was > 0.7 or < − 0.7 [80]. Collinear variables were considered redundant and removed from the analysis.

zOTU relative abundance, environmental metadata (e.g. average seawater temperature, average hours of daylight, Chl a, POC, NPOC and TSS concentration), season (summer versus winter) and sampling date were used for Bray-Curtis distance-based redundancy analysis (dbRDA) using the phyloseq package [76]. The significance of each response variable was confirmed with an analysis of variance (ANOVA) for the dbRDA (anova.cca() function in the vegan package [78]). Only significant (*p* value < 0.05) response variables were kept in the model. The explanatory value (in %) of significant response variables (e.g. environmental parameters, season and sampling date) was assessed with a variation partitioning analysis of the vegan package [78].

### Indicator value analysis

Indicator taxa were identified with the indicator value analysis (indicspecies package [37]) using the following thresholds: 1000 permutations, minimum specificity (At) and minimum sensitivity (Bt) set to 70% and *p* value ≤ 0.01.

### Random forest machine learning

Random forest machine learning was performed with the caret [81] and random forest package [82] in R [77]. zOTUs with non-zero abundance values in at least 10% of the samples (*n* = 48) were preselected and z-score standardised prior to model training. Random forest (with *n*_trees_ = 10,000) prediction error was measured with out-of-bag (OOB) error. Highest accuracy (lowest OOB estimated error rate) for classification was achieved with *m*_try_ = 100 zOTUS and for regression with *m*_try_ = 400 zOTUs. Importance of zOTUs was measured using the decrease in mean accuracy for classification and mean-squared error (% Inc. MSE) for regression.

## Additional file

Additional file 1: Supplementary figures and tables. Supplementary material contains additional information on the frequency of sampling (**Table S1**) and detailed statistical outputs (**Table S2-S8**). Furthermore, additional supplementary figures are illustrating alpha diversity measures of microbial communities associated with the distinct coral reef habitats (**Figure S1**), within and between time point similarities of microbial community composition (**Figure S2**), PCoA plots for sediment and seawater microbiomes (**Figure S3**), environmental variability at Geoffrey Bay (Magnetic Island) (**Figure S4**), collinearity of environmental metadata collected at Geoffrey Bay (Magnetic Island) (**Figure S5**), microbial indicator taxa, calculated with the Indicator Value analysis, for high and low temperature, Chla, POC and TSS concentrations (**Figure S6**), classification of seawater temperature based on Random Forest machine learning (**Figure S7**), Random Forest machine learning seawater temperature regression (**Figure S8**) and the relative fraction of stable and transient microbiomes associated with the distinct coral reef habitats (**Figure S9**). (DOCX 2596 kb)
