# Supplementary material for: Microbial indicators of environmental perturbations in coral reef ecosystems
Source: Microbiome. 2019 Jun 21;7:94. doi: 10.1186/s40168-019-0705-7 (PMC6588946; doi:10.1186/s40168-019-0705-7)
Supplement: Supplementary file 1 — Supplementary figures and tables. Supplementary material contains additional information on the frequency of sampling (Table S1) and detailed statistical outputs (Table S2-S8). Furthermore, additional supplementary figures are illustrating alpha diversity measures of microbial communities associated with the distinct coral reef habitats (Figure S1), within and between time point similarities of microbial community composition (Figure S2), PCoA plots for sediment and seawater microbiomes (Figure S3), environmental variability at Geoffrey Bay (Magnetic Island) (Figure S4), collinearity of environmental metadata collected at Geoffrey Bay (Magnetic Island) (Figure S5), microbial indicator taxa, calculated with the Indicator Value analysis, for high and low temperature, Chla, POC and TSS concentrations (Figure S6), classification of seawater temperature based on Random Forest machine learning (Figure S7), Random Forest machine learning seawater temperature regression (Figure S8) and the relative fraction of stable and transient microbiomes associated with the distinct coral reef habitats (Figure S9). (DOCX 2596 kb) [file 40168_2019_705_MOESM1_ESM.docx]

**Additional file 1**

**Microbial indicators of environmental perturbations in coral reef ecosystems**

**Table S1** Overview on the sample collection during February 2016 till May 2017 at the three sampling sites (Geoffrey Bay, Pioneer Bay, Channel) for each host/habitat. Numbers represent replicates collected per sampling event.


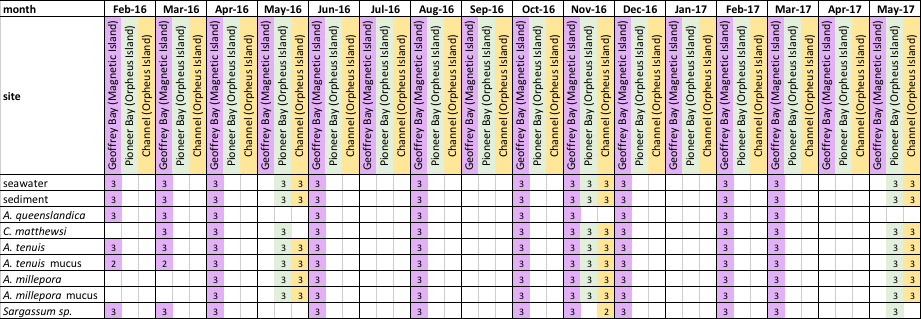


**Table S2** Statistical output of PERMANOVA (adonis, vegan package) testing the effect of host/habitat on the microbiome composition (10,000 permutations).

adonis(formula = d ~ Host, data = df, permutations = 10000, method = "bray")

**Df SumsOfSqs MeanSqs F.Model R2 Pr(>F)**

Host 8 48.362 6.0453 18.8 0.2879 9.999e-05 ***

Residuals 372 119.619 0.3216 0.7121

Total 380 167.982 1.0000

**Table S3** Statistical output of pairwise PERMANOVA (adonis, vegan package) testing compositional variation of microbial communities between host/habitat pairs (999 permutations, Bonferroni adjusted p values).

| **pairs** | **F.Model** | **R2** | **p.value** | **p.adjusted** |
| --- | --- | --- | --- | --- |
| Sediment vs Sargassum sp. | 13.86609753 | 0.146164941 | 0.001 | 0.036 |
| Sediment vs Coscinoderma matthewsi | 34.8432678 | 0.283640027 | 0.001 | 0.036 |
| Sediment vs Amphimedon queenslandica | 27.6842113 | 0.267005082 | 0.001 | 0.036 |
| Sediment vs Acropora tenuis | 18.14474956 | 0.161797584 | 0.001 | 0.036 |
| Sediment vs Acropora millepora | 17.97854581 | 0.169643258 | 0.001 | 0.036 |
| Sediment vs Acropora tenuis mucus | 20.66627379 | 0.183429105 | 0.001 | 0.036 |
| Sediment vs Acropora millepora mucus | 17.16713177 | 0.163236664 | 0.001 | 0.036 |
| Sediment vs seawater | 73.60303747 | 0.439150976 | 0.001 | 0.036 |
| Sargassum sp. vs Coscinoderma matthewsi | 19.72345911 | 0.208221483 | 0.001 | 0.036 |
| Sargassum sp. vs Amphimedon queenslandica | 15.49253476 | 0.197375901 | 0.001 | 0.036 |
| Sargassum sp. vs Acropora tenuis | 8.088787516 | 0.090794675 | 0.001 | 0.036 |
| Sargassum sp. vs Acropora millepora | 7.968698594 | 0.096044638 | 0.001 | 0.036 |
| Sargassum sp. vs Acropora tenuis mucus | 9.579134678 | 0.108142112 | 0.001 | 0.036 |
| Sargassum sp. vs Acropora millepora mucus | 7.42494157 | 0.090081248 | 0.001 | 0.036 |
| Sargassum sp. vs seawater | 44.1697348 | 0.352878712 | 0.001 | 0.036 |
| Coscinoderma matthewsi vs Amphimedon queenslandica | 32.90033449 | 0.319730102 | 0.001 | 0.036 |
| Coscinoderma matthewsi vs Acropora tenuis | 21.60797897 | 0.197138741 | 0.001 | 0.036 |
| Coscinoderma matthewsi vs Acropora millepora | 21.15260887 | 0.205061308 | 0.001 | 0.036 |
| Coscinoderma matthewsi vs Acropora tenuis mucus | 26.26867351 | 0.233980439 | 0.001 | 0.036 |
| Coscinoderma matthewsi vs Acropora millepora mucus | 21.21690133 | 0.205556465 | 0.001 | 0.036 |
| Coscinoderma matthewsi vs seawater | 89.53183211 | 0.504314246 | 0.001 | 0.036 |
| Amphimedon queenslandica vs Acropora tenuis | 14.67815749 | 0.161870928 | 0.001 | 0.036 |
| Amphimedon queenslandica vs Acropora millepora | 13.29072879 | 0.159570326 | 0.001 | 0.036 |
| Amphimedon queenslandica vs Acropora tenuis mucus | 11.09498399 | 0.130383525 | 0.001 | 0.036 |
| Amphimedon queenslandica vs Acropora millepora mucus | 9.565676514 | 0.120223656 | 0.001 | 0.036 |
| Amphimedon queenslandica vs seawater | 26.85720652 | 0.261111568 | 0.001 | 0.036 |
| Acropora tenuis vs Acropora millepora | 4.87949429 | 0.052535754 | 0.001 | 0.036 |
| Acropora tenuis vs Acropora tenuis mucus | 8.518986208 | 0.084750021 | 0.001 | 0.036 |
| Acropora tenuis vs Acropora millepora mucus | 6.727253834 | 0.071017089 | 0.001 | 0.036 |
| Acropora tenuis vs seawater | 42.14157098 | 0.30954227 | 0.001 | 0.036 |
| Acropora millepora vs Acropora tenuis mucus | 8.201091035 | 0.087059406 | 0.001 | 0.036 |
| Acropora millepora vs Acropora millepora mucus | 5.308371529 | 0.060800258 | 0.001 | 0.036 |
| Acropora millepora vs seawater | 39.17007406 | 0.308013299 | 0.001 | 0.036 |
| Acropora tenuis mucus vs Acropora millepora mucus | 1.812593058 | 0.020641607 | 0.004 | 0.144 |
| Acropora tenuis mucus vs seawater | 21.37760951 | 0.188552304 | 0.001 | 0.036 |
| Acropora millepora mucus vs seawater | 22.03462419 | 0.200251733 | 0.001 | 0.036 |

**Figure S1** Observed zOTU richness (left) and alpha diversity (right) of microbial communities associated with the distinct coral reef habitats. Alpha diversity was calculated with the Shannon Index (richness and evenness).

**Table S4** Statistical output of the TukeyHSD post hoc test (95% confidence interval) used to compare alpha diversity variances between coral reef habitats.

**diff lwr upr p adj**

Sargassum sp.-Sediment -2.92271459 -3.441838791 -2.4035904 0.0000000

Amphimedon queenslandica-Sediment -4.69996759 -5.243534391 -4.1564008 0.0000000

Coscinoderma matthewsi-Sediment -2.95723847 -3.450710862 -2.4637661 0.0000000

Acropora millepora-Sediment -4.04136337 -4.534835756 -3.5478910 0.0000000

Acropora millepora mucus-Sediment -2.80650334 -3.299975734 -2.3130310 0.0000000

Acropora tenuis-Sediment -4.05903506 -4.535774680 -3.5822954 0.0000000

Acropora tenuis mucus-Sediment -2.31162759 -2.793521308 -1.8297339 0.0000000

seawater-Sediment -2.93886053 -3.415600147 -2.4621209 0.0000000

Amphimedon queenslandica-Sargassum sp. -1.77725300 -2.358350350 -1.1961556 0.0000000

Coscinoderma matthewsi-Sargassum sp. -0.03452388 -0.569055699 0.5000079 0.9999999

Acropora millepora-Sargassum sp. -1.11864877 -1.653180593 -0.5841170 0.0000000

Acropora millepora mucus-Sargassum sp. 0.11621125 -0.418320572 0.6507431 0.9990218

Acropora tenuis-Sargassum sp. -1.13632047 -1.655444664 -0.6171963 0.0000000

Acropora tenuis mucus-Sargassum sp. 0.61108700 0.087225549 1.1349484 0.0093714

seawater-Sargassum sp. -0.01614593 -0.535270131 0.5029783 1.0000000

Coscinoderma matthewsi-Amphimedon queenslandica 1.74272912 1.184428841 2.3010294 0.0000000

Acropora millepora-Amphimedon queenslandica 0.65860422 0.100303946 1.2169045 0.0081029

Acropora millepora mucus-Amphimedon queenslandica 1.89346425 1.335163968 2.4517645 0.0000000

Acropora tenuis-Amphimedon queenslandica 0.64093253 0.097365731 1.1844993 0.0081513

Acropora tenuis mucus-Amphimedon queenslandica 2.38834000 1.840247164 2.9364328 0.0000000

seawater-Amphimedon queenslandica 1.76110706 1.217540264 2.3046739 0.0000000

Acropora millepora-Coscinoderma matthewsi -1.08412489 -1.593780988 -0.5744688 0.0000000

Acropora millepora mucus-Coscinoderma matthewsi 0.15073513 -0.358920966 0.6603912 0.9915912

Acropora tenuis-Coscinoderma matthewsi -1.10179659 -1.595268978 -0.6083242 0.0000000

Acropora tenuis mucus-Coscinoderma matthewsi 0.64561088 0.147157383 1.1440644 0.0020912

seawater-Coscinoderma matthewsi 0.01837795 -0.475094445 0.5118503 1.0000000

Acropora millepora mucus-Acropora millepora 1.23486002 0.725203928 1.7445161 0.0000000

Acropora tenuis-Acropora millepora -0.01767169 -0.511144083 0.4758007 1.0000000

Acropora tenuis mucus-Acropora millepora 1.72973577 1.231282278 2.2281893 0.0000000

seawater-Acropora millepora 1.10250284 0.609030450 1.5959752 0.0000000

Acropora tenuis-Acropora millepora mucus -1.25253171 -1.746004105 -0.7590593 0.0000000

Acropora tenuis mucus-Acropora millepora mucus 0.49487575 -0.003577744 0.9933292 0.0533513

seawater-Acropora millepora mucus -0.13235718 -0.625829572 0.3611152 0.9956668

Acropora tenuis mucus-Acropora tenuis 1.74740746 1.265513749 2.2293012 0.0000000

seawater-Acropora tenuis 1.12017453 0.643434911 1.5969142 0.0000000

seawater-Acropora tenuis mucus -0.62723293 -1.109126644 -0.1453392 0.0019332

**Table S5** Average alpha diversities based on Shannon Index for the distinct coral reef habitats (N= number of samples, sd= standard deviation, se = standard error, ci= 95% confidence interval).

**Habitat N Shannon sd se ci**

Sediment 48 7.371837 0.1883402 0.02718456 0.05468829

Sargassum sp. 35 4.449122 1.4363004 0.24277908 0.49338645

Amphimedon queenslandica 30 2.671869 0.7893556 0.14411595 0.29475021

Coscinoderma matthewsi 42 4.414598 0.3085990 0.04761785 0.09616621

Acropora millepora 42 3.330474 0.6596632 0.10178824 0.20556552

Acropora millepora mucus 42 4.565334 0.8739431 0.13485235 0.27233985

Acropora tenuis 48 3.312802 0.8167603 0.11788920 0.23716248

Acropora tenuis mucus 46 5.060209 0.8457770 0.12470301 0.25116476

seawater 48 4.432976 0.2036506 0.02939443 0.05913397

**Table S6** Average observed zOTU richness for the distinct coral reef habitats (N= number of samples, sd= standard deviation, se = standard error, ci= 95% confidence interval).

**Habitat N Observed sd se ci**

Sediment 48 2174.8125 202.93754 29.291511 58.92692

Sargassum sp. 35 690.2286 391.07958 66.104515 134.34054

Amphimedon queenslandica 30 256.3667 173.59435 31.693847 64.82120

Coscinoderma matthewsi 42 288.9762 67.18358 10.366652 20.93588

Acropora millepora 42 147.4048 48.60044 7.499211 15.14496

Acropora millepora mucus 42 323.2381 267.07492 41.210555 83.22641

Acropora tenuis 48 184.4167 162.22625 23.415342 47.10559

Acropora tenuis mucus 46 569.6957 363.28941 53.564101 107.88364

seawater 48 585.7083 54.47895 7.863359 15.81904

**Figure S2** Within and between time point similarities of microbial community composition using the ANOSIM R value as proxy (R = 0 indicates an even distribution of high and low dissimilarity ranks within and between time points and R = 1 indicates higher within than between sampling time points similarities).

Habitat

**Figure S3** PCoA plots for a) sediment and b) seawater illustrating compositional variations between samples collected at different sampling locations. The total variance (in percent) explained by each axis is indicated in parentheses.

**Figure S4** Environmental variability at the Geoffrey Bay (Magnetic Island) sampling location. Environmental metadata were standardized using the z-score standardisation method.

**Figure S5** Collinearity of environmental metadata collected at Geoffrey Bay (Magnetic Island) calculated with Pearson correlation (collinearity threshold: > 0.7 or < -0.7).

**Table S7** Statistical outputs of permutational ANOVAs for dbRDA analyses (anova.cca, vegan package) testing the significance of each predictor variable (10,000 permutations) on the microbiome composition for all habitats individually.

***a)* Seawater**

**Df SumOfSqs F Pr(>F)**

avg_temp 1 0.25153 10.0055 9.999e-05 ***

TSS 1 0.14231 5.6610 9.999e-05 ***

NPOC 1 0.19049 7.5777 9.999e-05 ***

Chla 1 0.15292 6.0831 9.999e-05 ***

avg_daylight 1 0.29859 11.8776 9.999e-05 ***

POC 1 0.27342 10.8763 9.999e-05 ***

Season 1 0.11235 4.4693 9.999e-05 ***

Sampling_Date 1 0.08958 3.5633 0.0005999 ***

Residual 21 0.52792

***b)* Sediment**

**Df Variance F Pr(>F)**

avg_temp 1 0.2714 1.4859 0.083916 .

TSS 1 0.3062 1.6762 0.059940 .

corse 1 0.6198 3.3929 0.001998 **

sand 1 0.4791 2.6224 0.006993 **

TOC_Sediment 1 0.5556 3.0414 0.001998 **

TON_Sediment 1 0.2299 1.2588 0.201798

Season 1 0.6750 3.6948 0.000999 ***

Residual 34 6.2110

***c) A. tenuis* mucus**

**Df SumOfSqs F Pr(>F)**

avg_temp 1 0.5119 1.8817 0.0021 **

TSS 1 0.9276 3.4098 9.999e-05 ***

NPOC 1 0.4017 1.4766 0.0287 *

Chla 1 0.3780 1.3895 0.0476 *

avg_daylight 1 0.4955 1.8213 0.0033 **

POC 1 0.4101 1.5076 0.0325 *

Season 1 0.4058 1.4917 0.0270 *

Sampling_Date 1 0.2561 0.9413 0.5629

Residual 19 5.1689

***d) A. millepora mucus***

**Df SumOfSqs F Pr(>F)**

avg_temp 1 0.5059 1.3576 0.0267973 *

TSS 1 0.6244 1.6755 0.0008999 ***

NPOC 1 0.4456 1.1959 0.1076892

Chla 1 0.5648 1.5156 0.0071993 **

avg_daylight 1 0.4616 1.2386 0.0701930 .

POC 1 0.4020 1.0788 0.2514749

Sampling_Date 1 0.4628 1.2419 0.0688931 .

***e) Sargassum sp.* biofilm**

**Df SumOfSqs F Pr(>F)**

avg_temp 1 0.8476 2.5646 0.000200 ***

TSS 1 0.4187 1.2668 0.115788

NPOC 1 0.5595 1.6929 0.008299 **

Chla 1 0.6494 1.9650 0.002100 **

avg_daylight 1 0.4930 1.4918 0.033797 *

POC 1 0.8226 2.4890 9.999e-05 ***

Season 1 0.5648 1.7089 0.006899 **

Sampling_Date 1 0.4994 1.5110 0.025697 *

Residual 21 6.9403

***f) A. tenuis* tissue**

**Df SumOfSqs F Pr(>F)**

avg_temp 1 0.5692 1.6364 0.0374 *

TSS 1 1.0185 2.9279 9.999e-05 ***

NPOC 1 0.4732 1.3604 0.1044

Chla 1 0.6047 1.7383 0.0232 *

avg_daylight 1 0.3951 1.1360 0.2606

POC 1 0.5607 1.6120 0.0380 *

Season 1 0.8261 2.3750 0.0020 **

Sampling_Date 1 0.2545 0.7317 0.8601

Residual 21 7.3048

***g) A. millepora* tissue**

**Df SumOfSqs F Pr(>F)**

avg_temp 1 0.4590 1.1638 0.19118

TSS 1 0.4239 1.0748 0.31617

NPOC 1 0.5043 1.2788 0.07799 .

Chla 1 0.3766 0.9550 0.53615

avg_daylight 1 0.5219 1.3234 0.07139 .

POC 1 0.3442 0.8728 0.72153

Season 1 0.4623 1.1722 0.17158

Residual 16 6.3102

***h) C. matthewsi***

**Df SumOfSqs F Pr(>F)**

avg_temp 1 0.23898 1.5206 0.0380 *

TSS 1 0.26510 1.6868 0.0124 *

NPOC 1 0.20436 1.3003 0.1221

Chla 1 0.20464 1.3021 0.1220

avg_daylight 1 0.40672 2.5879 9.999e-05 ***

POC 1 0.19280 1.2267 0.1721

Season 1 0.16873 1.0736 0.3488

Sampling_Date 1 0.15981 1.0169 0.4338

Residual 18 2.82895

***i) A. queenslandica***

**Df SumOfSqs F Pr(>F)**

avg_temp 1 0.3385 1.7979 0.1204

TSS 1 0.1998 1.0609 0.3259

NPOC 1 0.7094 3.7676 0.0109 *

Chla 1 0.1839 0.9768 0.3689

avg_daylight 1 0.2428 1.2892 0.2370

POC 1 1.3842 7.3512 0.0003 ***

Season 1 0.1909 1.0137 0.3422

Sampling_Date 1 0.1044 0.5542 0.7404

Residual 21 3.9543

**Table S8** Host-associated microbiomes and their sensitivity (% explained, Variation Partitioning Analysis, var.par, vegan package) to environmental parameters (permutational ANOVAs for dbRDA analyses (anova.cca, vegan package).

| **microbiome** | | **n** | | **% explained** | **environmental parameters**  (p-values: <0.05*, <0.01** and <0.001***) | |
| --- | --- | --- | --- | --- | --- | --- |
| *A. tenuis* mucus | 28 | | 17 | | temperature**, daylight**, TSS***, NPOC*, Chla* and POC* |  |
| *A. millepora* mucus | 24 | | 6 | | temperature*, TSS*** and Chla** |  |
| *Sargassum sp. biofilm* | 30 | | 14 | | temperature***, NPOC**, Chla**, daylight* and POC*** |  |
| *A. tenuis* tissue | 30 | | 14 | | Temperature*, TSS*** and Chla* |  |
| *A. millepora* tissue | 24 | | 0 | | n.s. |  |
| *C. matthewsi* | 27 | | 6 | | Temperature*, TSS* and daylight*** |  |
| *A. queenslandica* | 30 | | 19 | | NPOC* and POC*** |  |
| seawater | 30 | | 56 | | temperature***, daylight***, TSS***, NPOC***, Chla*** and POC*** |  |
| sediment | 30 | | 3 | | n.s. |  |

**Figure S6** Microbial indicator taxa, calculated with the Indicator Value analysis, for high and low temperature, Chla, POC and TSS concentrations. Each dot represents a unique zOTU.

**Figure S7** Classification of seawater temperature based on Random Forest machine learning. Highest accuracy was achieved with 100 randomly sampled zOTUs (left). Out-of-bag (OOB) error with 10,000 trees (right).

**Figure S8** Random Forest machine learning seawater temperature regression. RMSE (Root Mean Square Error), based on Out-of-Bag resampling, was lowest with 400 randomly sampled zOTUs (left). Out-of-bag (OOB) error with 10,000 trees (right).

**Figure S9** Stable *versus* transient microbiomes based on different abundance thresholds. Microbiomes were grouped based on their average relative abundances (>1%, 1-0.1% and <0.1%). The fractional contribution of stable (present in >80% of the samples) and transient (<50% of the samples) microbiomes are represented as boxplots.
